# Genetic Mapping of Natural Variation in Schooling Tendency in the Threespine Stickleback

**DOI:** 10.1534/g3.114.016519

**Published:** 2015-02-25

**Authors:** Anna K. Greenwood, Reza Ardekani, Shaugnessy R. McCann, Matthew E. Dubin, Amy Sullivan, Seth Bensussen, Simon Tavaré, Catherine L. Peichel

**Affiliations:** * Divisions of Basic Sciences and Human Biology, Fred Hutchinson Cancer Research Center, Seattle, Washington 98109; † Program in Molecular and Computational Biology, University of Southern California, Los Angeles, California 90089

**Keywords:** social behavior, evolution, fish, quantitative trait locus (QTL), repeatability

## Abstract

Although there is a heritable basis for many animal behaviors, the genetic architecture of behavioral variation in natural populations remains mostly unknown, particularly in vertebrates. We sought to identify the genetic basis for social affiliation in two populations of threespine sticklebacks (*Gasterosteus aculeatus*) that differ in their propensity to school. Marine sticklebacks from Japan school strongly whereas benthic sticklebacks from a lake in Canada are more solitary. Here, we expanded on our previous efforts to identify quantitative trait loci (QTL) for differences in schooling tendency. We tested fish multiple times in two assays that test different aspects of schooling tendency: 1) the model school assay, which presents fish with a school of eight model sticklebacks; and 2) the choice assay, in which fish are given a choice between the model school and a stationary artificial plant. We found low-to-moderate levels of repeatability, ranging from 0.1 to 0.5, in schooling phenotypes. To identify the genomic regions that contribute to differences in schooling tendency, we used QTL mapping in two types of crosses: benthic × marine backcrosses and an F2 intercross. We found two QTL for time spent with the school in the model school assay, and one QTL for number of approaches to the school in the choice assay. These QTL were on three different linkage groups, not previously linked to behavioral differences in sticklebacks. Our results highlight the importance of using multiple crosses and robust behavioral assays to uncover the genetic basis of behavioral variation in natural populations.

Social groups are found in a diverse range of animal taxa. The formation of social groups provides a number of benefits in the areas of predator avoidance, foraging, mate choice, and efficiency of movement (Krause and Ruxton 2002). However, there are also costs to group living, such as competition for food and mates (Krause and Ruxton 2002). Depending on the balance between these costs and benefits, animals can exhibit variation in the extent of group formation across species, populations of a single species, or even across the lifetime of an individual (Krause and Ruxton 2002). Although critical neurochemicals, hormones, and gene expression changes that mediate social behavior have been identified, the genetic basis for differences in the tendency to be social across individuals or species remains largely unstudied (Robinson *et al.* 2005, 2008; Hofmann *et al.* 2014). Knowledge of the genetic basis for behavioral differences will provide insight into both proximate and ultimate mechanisms for the evolution of social behavior (Hoekstra 2010).

Schooling behavior represents a fascinating example of a social grouping behavior that varies extensively both across and within species (Shaw 1978; Pitcher 1979, 1983; Pitcher and Parrish 1993). Threespine sticklebacks (*Gasterosteus aculeatus*) offer an interesting model system in which to explore variation in schooling behavior. Sticklebacks frequently are found in social groups in the wild (Wootton 1976), but the extent of social group formation varies in sticklebacks from different habitats (Vamosi 2002; Doucette *et al.* 2004; Wark *et al.* 2011; Kozak and Boughman 2012; Grobis *et al.* 2013; Di-Poi *et al.* 2014). For example, ancestral marine sticklebacks live in high-predation, open-water environments and exhibit a greater propensity to school in the wild and the lab (Wootton 1976; Wark *et al.* 2011; Di-Poi *et al.* 2014). By contrast, derived benthic sticklebacks from Paxton Lake, British Columbia, inhabit a heavily vegetated environment and tend to be solitary in the wild and the laboratory (Larson 1976; Vamosi 2002; Wark *et al.* 2011). Although the evolutionary forces that have led to reduced schooling in benthics are not known, it has been hypothesized that different predation regimes and increased access to shelter in the benthic environment might have resulted in relaxed selection for the maintenance of schooling behavior (Vamosi 2002; Wark *et al.* 2011).

Because schooling behavior emerges as a consequence of interactions with other individuals, it is has been difficult to study the genetic contributions to schooling behavior in an individual fish. Furthermore, genetic studies require assays with little environmental variance, but freely interacting social groups can vary considerably from trial to trial. To overcome these challenges, we previously developed an assay that uses a school of model fish to elicit naturalistic schooling behavior (Wark *et al.* 2011). The model school assay has been used successfully to measure schooling behavior in several species of fish (Lacasse and Aubin-Horth 2012; Grobis *et al.* 2013; Kowalko *et al.* 2013b; Di-Poi *et al.* 2014). We used this model school assay to show that sticklebacks from marine and benthic populations exhibit differences in two components of schooling behavior: they vary both in their tendency and ability to school (Wark *et al.* 2011). First, marine sticklebacks from a population in Japan have a stronger tendency to school, defined by a more rapid approach to the school and a longer time spent swimming with the school, than benthic sticklebacks from a freshwater lake in British Columbia (Wark *et al.* 2011). Second, marine sticklebacks show a more parallel body position with the models than benthic sticklebacks (Wark *et al.* 2011). Differences in schooling between these populations are heritable and not altered by social experience (Wark *et al.* 2011).

The model school assay further enabled us to examine the genetic basis for differences in schooling between marine and benthic sticklebacks (Greenwood *et al.* 2013). Using quantitative trait locus (QTL) mapping in a marine × benthic F2 intercross, we identified regions of the genome that underlie differences in schooling ability, measured by body positioning among the models (Greenwood *et al.* 2013). We further found evidence for genetic independence of the two components of schooling behavior, the tendency to school *vs.* schooling ability (Greenwood *et al.* 2013). Specifically, these variables are not correlated in F2 hybrids, and they do not map to the same regions of the genome. However, we did not identify any significant QTL for measures of schooling tendency, such as the time to initially approach or time spent with the model school (Greenwood *et al.* 2013).

In the present study, we sought to refine our experimental approach to investigate the genetic basis for differences in schooling tendency. There were several possible explanations for the lack of QTL in our first study, which we attempted to address in the current study. First, the lack of QTL might have resulted from a lack of power because few individuals in our first study showed the low amounts of schooling that typify benthic parental phenotypes (Wark *et al.* 2011; Greenwood *et al.* 2013). One explanation for this result is that reduced schooling might be due to the effects of homozygous recessive benthic alleles across multiple loci. Thus, to increase the number of loci with homozygous benthic alleles, we established backcrosses by crossing benthic × marine F1 hybrids to benthic fish. However, we also used a new benthic × marine F2 intercross to permit detection of loci with recessive marine effects.

Second, each F2 fish in the previous study was only tested once in the model school assay because of logistical constraints (Greenwood *et al.* 2013). Here, we tested each fish three times in the model school assay to obtain better estimates of the behavior of an individual fish by normalizing unpredictable environmental effects. Finally, we also tested the fish three times in a second “choice” assay, in which fish were given the choice between schooling and seeking shelter under an artificial plant. We developed this second assay because the school comprises the only form of shelter in the tank in the model school assay, so fish could follow the school as a result of an increased tendency to school or as a consequence of shelter-seeking behavior. Our previous work demonstrated that benthics show an even stronger reduction in schooling behavior compared to marines when tested in the choice assay (Wark *et al.* 2011). Together, these modifications enabled the discovery of several QTL associated with differences in the tendency to school between marine and benthic sticklebacks.

## Materials and Methods

### Crosses

Backcross and F2 intercross families were generated from benthic × marine F1 hybrids, which were produced by crossing a single Paxton benthic female with a single Japanese Pacific Ocean marine male. This F1 cross was independent of the cross in our previous study (Greenwood *et al.* 2013). Two backcross families were produced: a benthic female was crossed with an F1 male, yielding 176 fish (family 1); and an F1 female was crossed to a benthic male, yielding 200 fish (family 2). An F2 intercross was generated from the same F1 cross by mating F1 siblings. A single F1 pair was used to generate 64 fish, and an additional 31 fish were the result of random crosses of F1 individuals. The benthic fish were from a laboratory population originally derived from wild-caught fish from Paxton Lake on Texada Island, British Columbia, Canada. The marine male was from a laboratory population originally derived from wild-caught fish from the Bekanbeushi River in Akkeshi on Hokkaido Island, Japan.

Fish were housed in either 110-L or 473-L tanks containing 3.5 ppt saltwater (Instant Ocean, United Pet Group, Blacksburg, VA) at 16-hr light/ 8-hr dark cycles at approximately 16°. Fish were fed live brine shrimp nauplii and frozen mysis shrimp. All work was conducted in accordance with the Fred Hutchinson Cancer Research Center Institutional Animal Care and Use Committee (protocol #1575).

### Behavioral phenotyping

**Figure 1:**
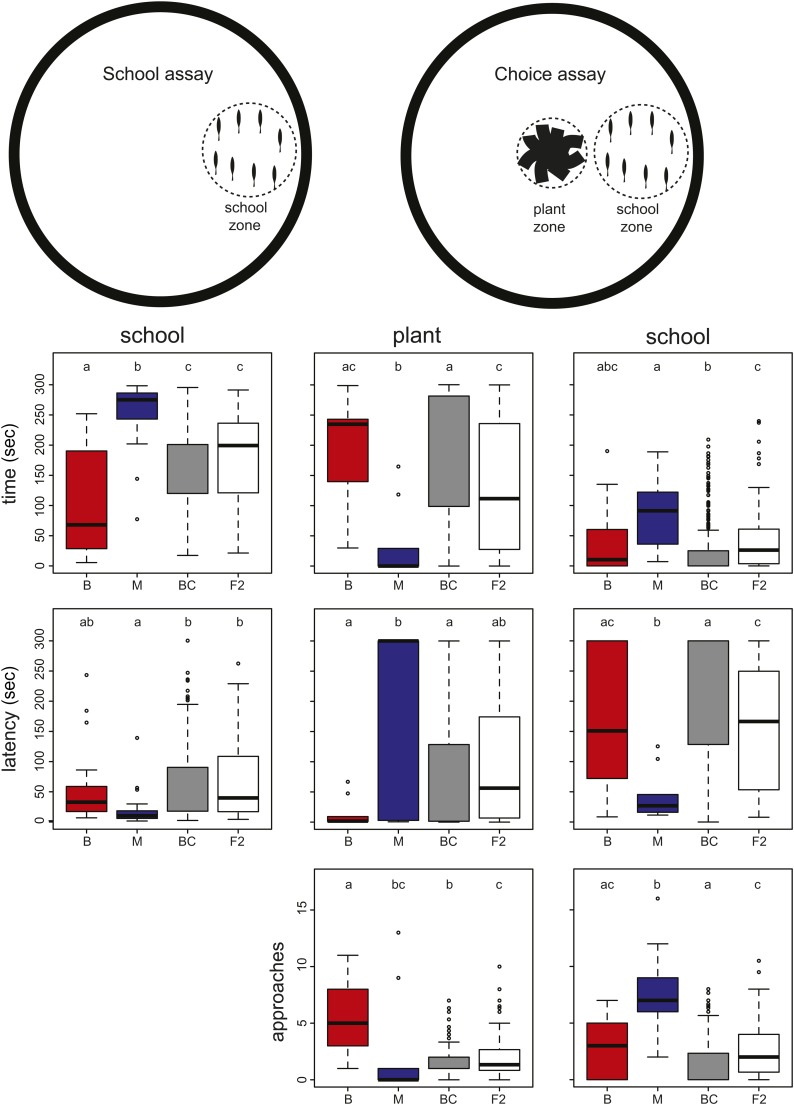
Phenotype distributions in parental lines and backcross and F2 hybrids. Schematics of the assay are depicted in the top panels, with the school and plant association zones indicated with dashed circles. Graphs show box plots for eight different phenotypic measures for benthic fish (B, red bars), marine fish (M, blue bars), backcross hybrids (BC, gray bars), and F2 hybrids (F2, white bars). Time spent schooling and latency to school were measured in the school-only assay. Six phenotypes were measured in the choice assay: time spent with plant and school, latency to join plant and school, and approaches to plant and school. Box plots show the median and 25% and 75% quartiles, whiskers show the 1.5× interquartile range, and outliers are shown in open circles. Significance groups determined by analysis of variance and Tukey’s HSD *post hoc* tests are indicated by lowercase letters.

Fish were tested in two different assays: the model school-only assay and the school *vs.* plant choice assay (see Figure 1). Assay construction was previously described in detail (Wark *et al.* 2011). In summary, both assays took place in a 61-cm diameter white tank filled with water to a height of 8 cm. In the school-only assay, fish were presented with a school of eight model sticklebacks, which were cast from a benthic × marine F2 hybrid that was 5 cm in length. The model school was moved counterclockwise in a circle at the outer edge of the tank at a speed of 5.5 rotations per minute. In the choice assay, the model school was positioned and moved in the same way, and an artificial plant made from strips of black garbage bags was placed in the center of the tank.

Fish were tested as nonreproductive juveniles, with an average size of 4.6 cm at the start of testing. Fish from each family were tagged with fluorescent elastomer (Northwest Marine Technology, Shaw Island, WA) and then separated into 110-L tanks containing a maximum of 16 fish. Fish were in their new tanks a minimum of 1 wk before behavioral trials began. Fish were tested three times each in the school-only assay and three times each in the choice assay. Fish were first tested in the school-only assay, and then trials alternated between the choice assay and the school assay, with a minimum of 1 wk between trials.

All fish from a tank were tested on the same day. Fish were moved into 950-mL holding chambers and left in isolation for a minimum of 90 min. An individual fish was then netted from the isolation chamber and placed into the assay tank. Fish were given a 5-min acclimation with the model school in a stationary position after which time the motor was turned on remotely for a 5-min trial with the moving school. For the choice assays, the plant was in position at the start of the acclimation. Two tanks of fish typically were tested per day and the water in the assay tank was changed in between groups.

Five different experimenters tested fish from the backcrosses and two of these experimenters also tested fish from the F2 intercross. Some backcross fish (n = 21) were excluded from the experiment because of loss of elastomer tags or death. Individual trials were excluded when necessary because of experimenter error (*e.g.*, failure to correctly position plant) and/or technical issues with the apparatus (*e.g.*, breakage of belt controlling school movement).

Videos of each trial were recorded using a SONY HC9 digital camcorder (SONY, San Diego, CA). Videos were digitized with the use of iMovie software (Apple, Inc., Cupertino, CA). Videos were then analyzed for behavioral variables. The school-only trials were scored by the use of two methods. The time spent with the school, defined by a fish swimming within one body length of the school (Wark *et al.* 2011), was determined with custom-designed tracking software (Ardekani *et al.* 2013). The latency to approach the school was determined manually by recording the frame number at which fish first joined the school. Fish that never approached the school were given a latency of 300 sec, *i.e.*, the total length of the trial. Number of approaches to the school was not determined in the school-only trials. Choice trials were scored by manually recording frame numbers when a fish entered or exited the school or plant zones. The school zone was defined as being within one body length from the models, and the plant zone was defined as being within one body length from the plant (Wark *et al.* 2011). Latency to approach the plant and/or school, time spent with the plant and/or school, and number of approaches to the plant and/or school were then determined. Fish that never approached the plant and/or school were given a latency of 300 sec, *i.e.*, the total length of the trial.

### QTL analysis

Backcross (162 fish from family 1 and 193 fish from family 2) and F2 intercross (95 fish) individuals were genotyped using custom-made Golden Gate (Illumina, San Diego, CA) single-nucleotide polymorphism (SNP) arrays (Jones *et al.* 2012). Genotypes were determined with GenomeStudio Software (Illumina). There were 279 and 260 informative SNP markers for the backcrosses and F2 intercross, respectively; 197 markers were shared across the backcrosses and F2 intercross. JoinMap 4.1 (Van Ooijen 2011) was used to construct linkage maps using the regression mapping algorithm. A single linkage map was generated from the combined genotypes of both backcross families. All genotype and phenotype data for these crosses are provided in File S1 and File S2.

QTL analysis was performed in R/qtl (Broman and Sen 2009). Analyses for the F2 intercross and backcrosses were performed separately. Both backcross families were combined for QTL mapping, and cross direction (family) was included as a covariate. There were no substantive differences in QTL results when sex was included as a covariate, so it was omitted from the final model. Logarithm of odds (LOD) significance thresholds and *P* values for each trait were determined by permutation testing (1000 permutations). Significant QTL were above the genome-wide threshold (α = 0.05). The fitqtl function was used to estimate the percent variance explained by the peak marker for each QTL. Additivity and dominance were estimated using the effectscan function. To estimate the impact of a lower sample size on the power to detect QTL in the F2 intercross, we calculated LOD scores for 10,000 random draws of n = 95 fish from the entire backcross (n = 355) and asked whether any LOD scores were above the LOD significance threshold calculated for a backcross of n = 95.

### Statistics

All statistical analyses were performed in R (http://www.r-project.org/). Differences between phenotype distributions were assessed with analysis of variance (ANOVA) and Tukey’s HSD *post hoc* tests. Data for parental lines were all taken from a previous publication and presented here for comparison to the backcrosses and F2 intercross data (Wark *et al.* 2011). Correlations between phenotypes were calculated using Spearman’s Rho. The significance threshold was corrected for multiple comparisons using the false discovery rate approach (Benjamini *et al.* 2001). ANOVA with Tukey’s HSD *post hoc* tests was used to calculate differences in mean phenotype across populations. Single-marker ANOVA was used to test for an association between time spent schooling in the F2 cross and the locus on linkage group (LG) 21 identified in the backcrosses (see *QTL mapping of schooling behavior in genetic crosses*). Repeatability was calculated using the rptR package in R (Nakagawa and Schielzeth 2010). Linked scale repeatability for count data (number of approaches) was calculated using a generalized linear mixed-effects model (poisGLMM.add). Latency and time data were first log-transformed, and then repeatability was calculated using a linear mixed-effects model (remlLMM).

## Results and Discussion

### Phenotypic analysis of genetic crosses

#### Distribution of schooling phenotypes:

To investigate the genetic basis for differences in schooling behavior, we first compared the phenotype distributions of parental and hybrid cross individuals in the school-only and school *vs.* plant choice assays. In the school-only assay, marines spent significantly more time with the school and showed a tendency to join the school with a shorter latency (Figure 1). In the choice assay, benthics spent significantly more time with the plant than marines (Figure 1).

Backcross and F2 intercross hybrids typically showed wide phenotype distributions, suggesting that these traits are likely controlled by multiple genes (Figure 1). For all but one variable, the average behavior of hybrid crosses was either intermediate between the parental population means, or not statistically different from benthic values (Figure 1). These results suggest that benthic alleles are typically dominant or semi-dominant for these behaviors. Interestingly, this result is contrary to our initial hypothesis that benthic alleles might be recessive (see Introduction). The exception to this trend was the number of approaches to the plant in the choice assay, in which hybrid behavior was statistically indistinguishable from that of marine fish (Figure 1).

#### Repeatability of schooling phenotypes:

We next calculated the repeatability of phenotypic measures for the backcrosses and F2 intercross (Table 1), to understand the extent of environmental and genetic variance contributing to these traits. Repeatability tended to be higher for the variables from the choice trials (average = 0.42; Table 1) than the school-only trials (average = 0.19; Table 1). The greatest average repeatability was for the approaches to the school in the choice assay (Table 1). In addition, repeatability was greater for the F2 intercross (average = 0.42) than the backcrosses (average = 0.31) for most variables. Lower repeatability estimates in the backcrosses could reflect greater environmental variance caused by procedural differences (Lynch and Walsh 1998). Backcross individuals were tested by between one and three different experimenters (average = 2.2), whereas the behavioral trials for the F2 individuals were conducted such that each F2 individual was handled by a single experimenter.

**Table 1:** Repeatability of phenotypes

Phenotype	Backcrosses	F2 Intercross
School: time with school	0.132 ± 0.03*^a^*	0.184 ± 0.07
School: latency to school	0.146 ± 0.03*^a^*	0.306 ± 0.07
Choice: time with school	0.455 ± 0.04	0.452 ± 0.06
Choice: latency to school	0.296 ± 0.04	0.487 ± 0.06
Choice: approaches to school	0.446 ± 0.04	0.536 ± 0.08*^a^*
Choice: time with plant	0.402 ± 0.03	0.509 ± 0.06
Choice: latency to join plant	0.370 ± 0.04	0.480 ± 0.07
Choice: approaches to plant	0.205 ± 0.04	0.393 ± 0.08

Repeatability estimates ± SE of the estimate are reported for each phenotype for the backcrosses and F2 intercross.

a Indicates phenotypes for which a significant QTL was identified.

#### Correlations among schooling phenotypes:

To gain insight into the relationship between the phenotypes measured in the two behavioral assays we performed correlation analysis (Table 2). The strength and direction of correlation (measured by Spearman’s Rho) between variables was generally similar in the backcrosses and F2 intercross, although more correlations reached statistical significance in the backcrosses due to the larger sample size. School-only variables were highly correlated with one another (Table 2; backcross: Rho = −0.68; F2: Rho = −0.77). Most phenotypes from the choice trials also were strongly intercorrelated, with the exception of the number of approaches to the plant, which was weakly correlated with most other variables (we define “weak” correlations as those with Rho < 0.3). Interestingly, the number of approaches to the plant also showed a different inheritance pattern from all other traits, with hybrid behavior being similar to marine behavior (see *Distribution of schooling phenotypes*).

**Table 2:** Phenotypic correlations

	School: Latency to Join School		Choice: Time With School		Choice: Latency to Join School		Choice: Approaches to School		Choice: Time With Plant		Choice: Latency to Join Plant		Choice: Approaches to Plant	
	BC	F2	BC	F2	BC	F2	BC	F2	BC	F2	BC	F2	BC	F2
School: time with school	−0.68*^a^*	−0.77*^a^*	−0.12*^a^*	0.10	0.13*^a^*	0.08	−0.14*^a^*	−0.04	0.32*^a^*	0.40*^a^*	−0.34*^a^*	−0.43*^a^*	0.01	0.20
School: latency to join school			0.06	−0.26*^a^*	−0.09	0.16	0.07	−0.18	−0.22*^a^*	−0.20	0.25*^a^*	0.23*^a^*	−0.04	−0.20
Choice: time with school					−0.89*^a^*	−0.80*^a^*	0.95*^a^*	0.85*^a^*	−0.73*^a^*	−0.60*^a^*	0.55*^a^*	0.41*^a^*	0.16*^a^*	0.12
Choice: latency to join school							−0.92*^a^*	−0.89*^a^*	0.73*^a^*	0.70*^a^*	−0.58*^a^*	−0.50*^a^*	−0.12*^a^*	−0.13
Choice: approaches to school									−0.75*^a^*	−0.67*^a^*	0.57*^a^*	0.44*^a^*	0.15*^a^*	0.22
Choice: time with plant											−0.88*^a^*	−0.83*^a^*	0.15*^a^*	0.34*^a^*
Choice: latency to join plant													−0.33*^a^*	−0.54*^a^*

Correlations between phenotypic variables from school-only (School) and choice (Choice) assay trials for backcrosses (BC) and F2 intercross (F2). Spearman’s Rho is reported.

a Indicates correlations with *P*-values below the FDR corrected thresholds of *P* < 0.04 for BC and *P* < 0.03 for F2.

In contrast, most behavioral measures across the school-only and choice assays were weakly correlated (Rho < 0.3), with two exceptions. There was a negative correlation between time spent with the school in the school-only assay and the latency to join the plant in the choice assay (backcross: Rho = −0.34; F2: Rho = −0.43). In addition, there was a positive correlation between time spent with the school in the school-only assay and the total time spent with the plant in the choice assay (backcross: Rho = 0.32; F2: Rho = 0.40). The latter correlation is surprising if time with the school exclusively measures schooling tendency, and suggests that increased time with the school in the school-only assay may reflect shelter-seeking behavior in some fish. In summary, because most behavioral measures from the school-only and choice assays were not strongly correlated in hybrid fish, we conclude that these assays measure different aspects of schooling behavior.

### QTL mapping of schooling behavior in genetic crosses

To identify the genomic regions that are associated with differences in schooling behavior between marine and benthic sticklebacks, we performed QTL analysis. We genotyped fish with a genome-wide panel of SNP markers and generated separate linkage maps for each cross type. Markers from each of the 21 chromosomes in threespine stickleback are represented in both the backcross and F2 intercross linkage maps. For the backcross, the final linkage map contained 22 LGs, with a total of 1322 cM length and an average spacing of 5.1 cM between markers. The linkage map for the F2 intercross comprised 25 LGs, with a total of 1081 cM length and an average marker spacing of 4.6 cM.

#### School-only assay:

**Figure 2:**
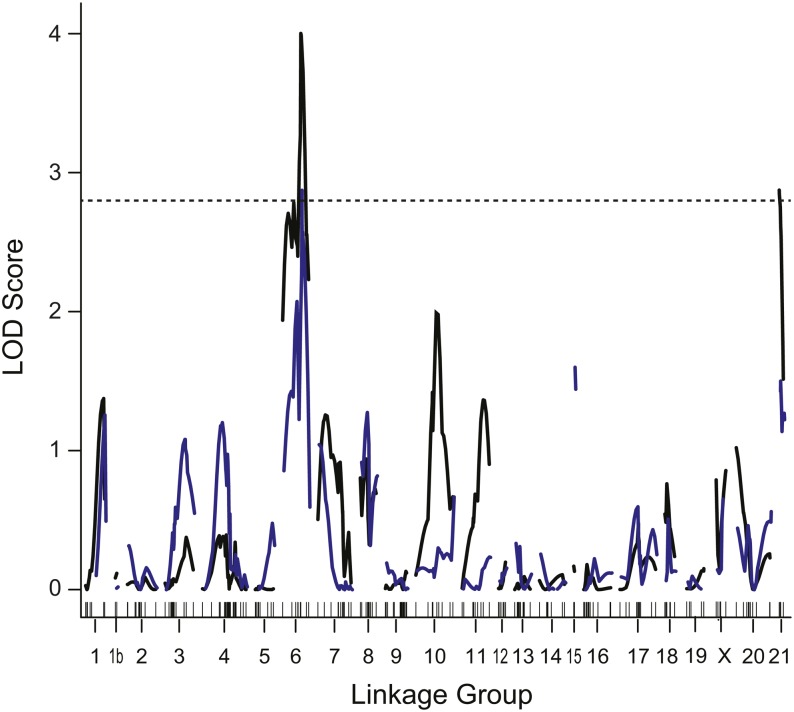
QTL associated with the school-only assay. LOD score as a function of distance in cM across each linkage group is shown for the backcrosses; there were no significant QTL in the F2 intercross. LOD scores for time with school are shown by a black line, and latency to school by a blue line. The horizontal dashed line indicates genome-wide significance at *P* < 0.05. QTL, quantitative trait locus; LOD, logarithm of odds.

In the F2 cross, there were no QTL that met genome-wide significance thresholds for variables in the school-only trials (data not shown). In the backcrosses, we identified two significant QTL for variables in the school-only trials, one on LG6 and one on LG21 (Figure 2 and Table 3). Both the time spent with the school and the latency to join the school mapped to a region on LG6. Surprisingly, an increased number of benthic alleles at this QTL led to more time with the school and a shorter latency to school, which is the opposite direction from parental phenotypes (Table 3). This negative QTL may be evidence that the school-only assay is not accurately separating schooling from shelter-seeking behavior. It is possible that benthic alleles at this locus on LG6 actually induce shelter-seeking behavior, which is measured as time spent with the school in the school-only assay. In the future, it would be interesting to further modify the assay, for instance using predator exposure, to better distinguish shelter-seeking from schooling behavior.

**Table 3:** Significant QTL

Assay	Phenotype	Cross	LG	Marker	Position	LOD	*P*	PVE	BB	MB	MM
School	Time with school	BC	6	chrVI:14131973	48.9	4	0.007	5.15	174 ± 4	148 ± 4	
School	Time with school	BC	21	chrXXI:774193	0	2.86	0.048	3.66	150 ± 4	171 ± 4	
School	Latency to join school	BC	6	chrVI:13775642	48.7	2.87	0.045	3.65	49 ± 4	69 ± 4	
Choice	Approaches to school	F2	10	chrX:10415917	13.14	4.87	0.006	21	1.3 ± 0.4	2.3 ± 0.3	4.4 ± 0.4

For each QTL the phenotype, cross in which it was detected, linkage group (LG), marker at peak, position in centimorgans (cM), logarithm of odds (LOD) score, *P*-value (*P*), percent variance explained (PVE), and phenotypic means ± SE are reported. QTL, quantitative trait locus; BB, homozygous benthic; MB, heterozygous; MM, homozygous marine.

Alternatively, the negative effect QTL on LG6 could truly reflect phenotypic effects in the opposite direction from parental means. Such “negative QTL” frequently are found in QTL analyses (Gardner and Latta 2007). Negative QTL for a particular phenotype can result when selection acts on QTL with pleiotropic effects (Griswold and Whitlock 2003). Negative effect QTL for time spent with the school could also reflect lack of directional selection for this phenotype (Orr 1998; Rieseberg *et al.* 2002). Processes such as drift or relaxed selection could result in polymorphism among benthic individuals. This in turn could lead to founder differences among crosses. Possibly reflecting variation among benthic individuals, the LG6 QTL was not identified in the F2 intercross and was stronger in one of the two backcross families (average effect of benthic allele substitution on time spent schooling was 21 sec in family 1 and 30 sec in family 2).

**Figure 3:**
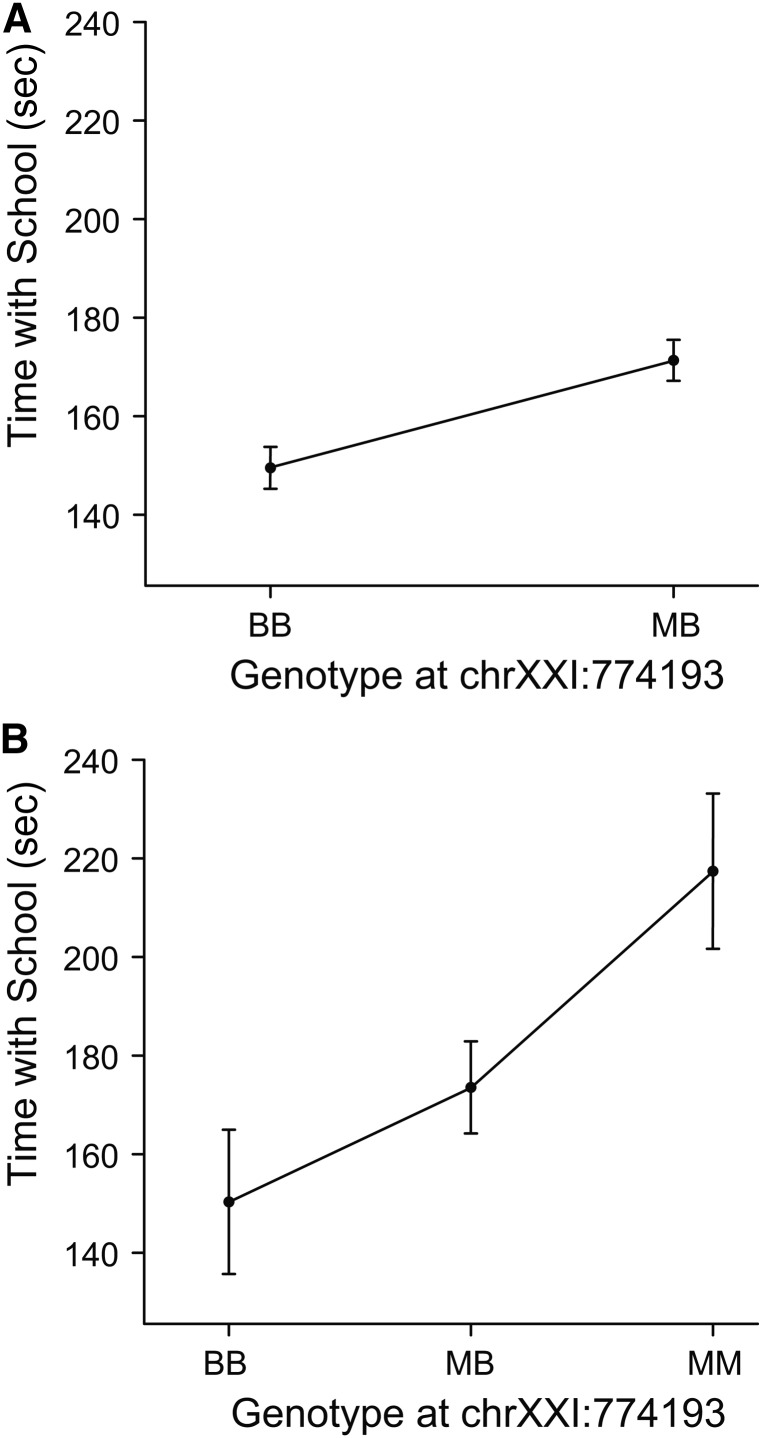
Parallel QTL on LG21 for time spent with school in the backcrosses and F2 intercross. Graphs show time with school as a function of allelic combinations at marker chrXXI:774193 in the backcrosses (A) and F2 intercross (B). There is a significant effect of genotype in both the backcrosses and F2 intercross (*P* < 0.05).

By contrast, benthic alleles at the QTL on LG21 were associated with phenotypic effects in the expected direction: backcross fish with two benthic alleles spent less time with the school (Figure 2 and Table 3). Although there was no significant genome-wide QTL on LG21 in the F2 intercross, we asked whether there was evidence for a genetic association in this region. Indeed, using single-marker analysis, the same marker (chrXXI:774193) was significantly associated with time spent with the school in the F2 intercross (Figure 3; F_1,93_ = 9.3, *P* < 0.003).

The lack of detectable QTL for the school-only assay in the F2 intercross could in part stem from low power to detect QTL of small effect in a cross of 95 individuals (Doerge 2002). It is difficult to fully assess the impact of sample size differences as backcross and F2 intercross designs have different power to detect QTL (Beavis 1998). However, to approximate the effect of sample size, we randomly drew 10,000 samples of 95 from the larger backcross population (n = 355). The results of this subsampling suggest that a lower sample size does impact our ability to detect QTL in the F2 intercross, as a significant QTL on LG21 was never detected and a QTL on LG6 was detected in only 2 of 10,000 samples of n = 95 in the backcross.

In our previous QTL analysis of schooling behavior using the school-only assay in an F2 intercross, we identified a “suggestive” QTL for latency to join the school on LG20, which met a genome-wide significance threshold of *P* < 0.1 (Greenwood *et al.* 2013). We did not detect linkage to LG20 in either cross in the current study. This could be attributable to procedural differences: as described in the Introduction fish in our previous QTL study were only tested in a single trial. Alternatively, this could reflect within-population allelic variation, as the previous and current studies used different benthic and marine individuals to establish genetic crosses.

#### Choice assay:

**Figure 4:**
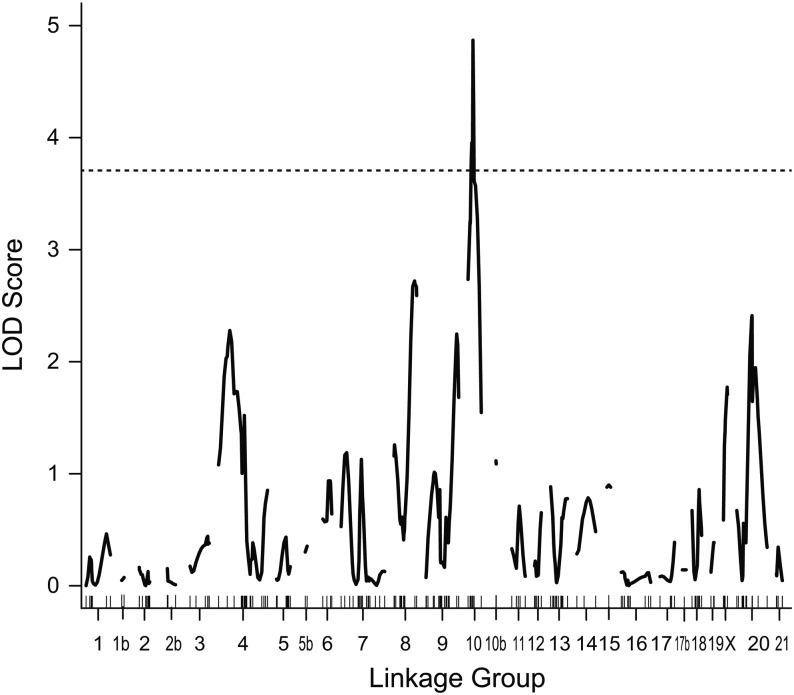
QTL associated with the choice assay. LOD score as a function of distance in cM across each linkage group is shown for the F2 intercross; there were no significant QTL in the backcrosses. LOD scores for number of approaches to the school are shown by a black line; there were no significant QTL for other phenotypic measures. The horizontal dashed line indicates genome-wide significance at *P* < 0.05.

**Figure 5:**
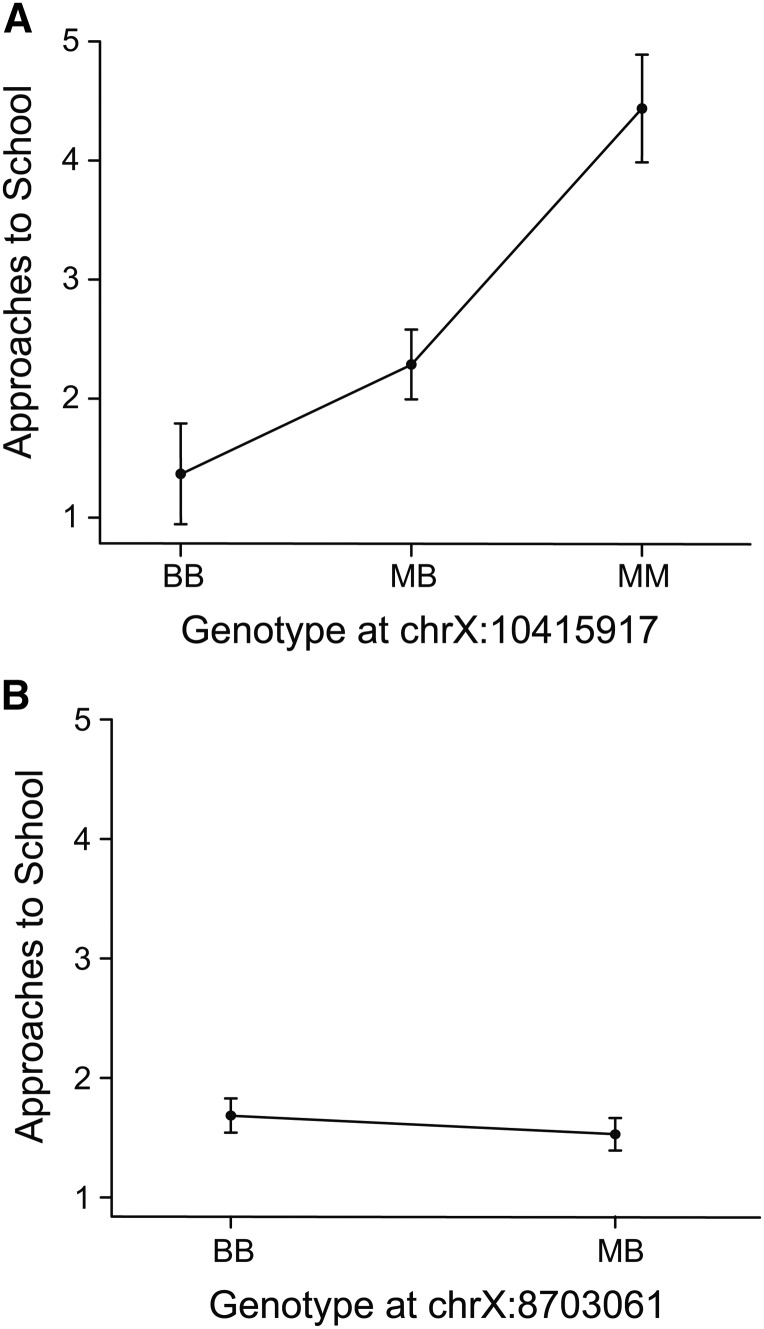
Lack of parallel QTL on LG10 for number of approaches to the school in the backcrosses and F2 intercross. Graphs show number of approaches to the school in the choice assay as a function of allelic combinations at marker chrX:10415917 in the F2 intercross (A) and at the closest marker (chrX:8703061) in the backcrosses (B). There is a significant effect of genotype in the F2 intercross (*P* < 0.05), but no effect in the backcrosses (*P* > 0.05).

We next performed QTL mapping of behaviors in the choice assay. There were no QTL that reached genome-wide significance in the backcrosses. However, in the F2 intercross, there was a significant QTL on LG10 for the number of approaches to the school in the choice trials (Figure 4). Benthic alleles at this locus acted to decrease approaches to the school (Table 3), with a mixture of additive and dominant effects (estimated effects: additive: 1.42, dominance: −0.23). We found no significant genotype-phenotype associations in this region in the backcrosses, even using single-marker analysis (Figure 5). The lack of a parallel QTL in the backcrosses and F2 intercross could in part result from the fact that benthic alleles at this locus were partially dominant, and dominant benthic effects would not be detectible in the backcrosses.

### Prospects for QTL mapping of behavioral variation in natural populations

We identified several QTL for different aspects of schooling behavior in two independent assays. We detected QTL for time spent schooling and latency to school in the school-only assay, although we had previously failed to detect QTL for these traits in our initial study (Greenwood *et al.* 2013). This finding suggests that our strategy to use a backcross design and test fish multiple times improved our ability to detect QTL. We also identified a QTL for number of approaches to the school in the choice assay. The QTL we detected were of relatively small effect size, which is consistent with the genetic architecture for complex behaviors in other species (Flint and Mott 2001; Anholt and Mackay 2004; Bendesky and Bargmann 2011; Weber *et al.* 2013). In fact, these modest effect sizes are likely overestimates, due to the relatively small sample size of our crosses (Beavis 1998). This tendency to overestimate is seen in the higher QTL effect size estimates in the F2 intercross (n = 95) than the backcross (n = 355) (Table 3).

There were five phenotypes in the choice assay for which we did not detect any QTL (Table 1). This lack of QTL likely results from low power to detect QTL due to sample size limitations (Doerge 2002). However, even with this modest sample size, we can exclude the chance that a single gene of large effect modulates these behaviors. The lack of detection of QTL for these phenotypic measures does not imply that there is not a genetic basis for these behaviors; indeed, many of these traits showed moderately high repeatability, which can reflect levels of heritability. Unexpectedly, in our study there was no clear correlation between trait repeatability and QTL detection. We identified QTL for both the trait with the lowest repeatability (0.13, time with school in the school-only assay) and the trait with the greatest repeatability (0.54, number of approaches to the school in the choice assay). Typically, greater repeatability (and heritability) is associated with increased power to detect QTL (Staub *et al.* 1996; Carlborg *et al.* 2005), although this is not always the case (Saintagne *et al.* 2004).

Despite the promise of genetic linkage mapping for identifying the genetic contributions to behavior (Fitzpatrick *et al.* 2005; Pollen and Hofmann 2008; Bendesky and Bargmann 2011), only a limited, but growing, number of studies have reported QTL for behavioral variation in natural populations of vertebrates (Koide *et al.* 2000; Yalcin *et al.* 2004; Takahashi *et al.* 2006; Wright *et al.* 2006; Rogers and Bernatchez 2007; Kitano *et al.* 2009; Wirén and Jensen 2011; Kukekova *et al.* 2011; Yoshizawa *et al.* 2012; Greenwood *et al.* 2013; Kowalko *et al.* 2013a,b; Weber *et al.* 2013; Christensen *et al.* 2014; Laine *et al.* 2014). It is likely that some linkage analysis studies of behavior result in no QTL, and thus remain unpublished, even when heritability of behavior is known. Even for cases in which genomic loci for natural behavioral variation have been detected, only a single causal gene has been identified in vertebrates (Yalcin *et al.* 2004). Thus, behavioral genetics remains a tantalizing but challenging area of inquiry. Reasons for this difficulty include a multigenic architecture and high sensitivity to environmental influence (Flint and Mott 2001; Flint 2003; Anholt and Mackay 2004; Bendesky and Bargmann 2011). Our data suggest that the use of multiple crosses with large numbers of individuals coupled with robust behavioral assays is key to the identification of the genetic changes that underlie behavioral variation in natural populations, which will ultimately shed light on the evolution of behavior.
